# Suppression of Proliferation of Human Glioblastoma Cells by Combined Phosphodiesterase and Multidrug Resistance-Associated Protein 1 Inhibition

**DOI:** 10.3390/ijms22189665

**Published:** 2021-09-07

**Authors:** Liliya Kopanitsa, Maksym V. Kopanitsa, Dewi Safitri, Graham Ladds, David S. Bailey

**Affiliations:** 1 IOTA Pharmaceuticals Ltd., St John’s Innovation Centre, Cowley Road, Cambridge CB4 0WS, UK; 2 The Francis Crick Institute, 1 Midland Road, London NW1 1AT, UK; maksym.kopanitsa@crick.ac.uk; 3 Department of Pharmacology, University of Cambridge, Tennis Court Road, Cambridge CB2 1PD, UK; ds791@cam.ac.uk (D.S.); grl30@cam.ac.uk (G.L.)

**Keywords:** glioblastoma, drug combination, multidrug resistance-associated protein 1, phosphodiesterase inhibitor, proliferation

## Abstract

The paucity of currently available therapies for glioblastoma multiforme requires novel approaches to the treatment of this brain tumour. Disrupting cyclic nucleotide-signalling through phosphodiesterase (PDE) inhibition may be a promising way of suppressing glioblastoma growth. Here, we examined the effects of 28 PDE inhibitors, covering all the major PDE classes, on the proliferation of the human U87MG, A172 and T98G glioblastoma cells. The PDE10A inhibitors PF-2545920, PQ10 and papaverine, the PDE3/4 inhibitor trequinsin and the putative PDE5 inhibitor MY-5445 potently decreased glioblastoma cell proliferation. The synergistic suppression of glioblastoma cell proliferation was achieved by combining PF-2545920 and MY-5445. Furthermore, a co-incubation with drugs that block the activity of the multidrug resistance-associated protein 1 (MRP1) augmented these effects. In particular, a combination comprising the MRP1 inhibitor reversan, PF-2545920 and MY-5445, all at low micromolar concentrations, afforded nearly complete inhibition of glioblastoma cell growth. Thus, the potent suppression of glioblastoma cell viability may be achieved by combining MRP1 inhibitors with PDE inhibitors at a lower toxicity than that of the standard chemotherapeutic agents, thereby providing a new combination therapy for this challenging malignancy.

## 1. Introduction

Glioblastoma multiforme (GBM) is the most common and aggressive brain tumour in adults, and its treatment options are limited [1]. The standard therapy is surgical resection followed by radiotherapy and concomitant chemotherapy with temozolomide [2]. Despite recent progress in understanding the disease, the 2-year and 5-year survival rates of patients with GBM remain poor, at only 27 and 5%, respectively [3,4]. Glioblastoma exhibits high invasive potential and inherent resistance to the currently available treatments; therefore, novel targeted therapeutic strategies are urgently needed [5,6].

Inhibition of cyclic nucleotide-specific phosphodiesterases (PDEs) is one promising avenue for GBM treatment. These enzymes catalyse the hydrolysis of the important intracellular second messengers cyclic adenosine monophosphate (cAMP) and cyclic guanosine monophosphate (cGMP), thereby disrupting many of the biochemical cascades involved in cancer [7]. It is known that glioblastoma cells are sensitive to intracellular cAMP levels, and their apoptosis can be triggered by cAMP elevation [8,9,10,11]. Furthermore, the expression of several PDEs is upregulated in glioblastoma cells [12,13,14], and the specific isoforms PDE1C, PDE4A, PDE4B, PDE4D and PDE5 have been suggested as therapeutic targets in glioblastoma [9,10,13]. Multiple brain-penetrant inhibitors of various PDEs are currently in clinical trials for neurodegenerative conditions such as Alzheimer’s disease, Parkinson’s disease, and Huntington’s disease [15], and these are available for repurposing in glioblastoma treatment.

In addition to PDEs, the levels of cAMP and cGMP are also regulated by the activities of multidrug resistance-associated proteins (MRPs) [16], and exposure to MRP inhibitors was shown to sensitise glioblastoma cells to chemotherapeutic drugs [17,18,19,20].

To assess the pharmacological inhibition of PDEs as a potential glioblastoma treatment, we examined the effects of 28 selected PDE inhibitors, covering the entire range of PDE isoforms, on cell proliferation and cAMP and cGMP levels in the human glioblastoma cell lines U87MG, A172 and T98G. We also investigated the effects of the co-treatment of glioblastoma cells with PDE and MRP inhibitors to find drug combinations with maximal antiproliferative action.

## 2. Results

### 2.1. Effects of PDE Inhibitors on the Viability of Glioblastoma Cell Lines

**Figure 1:**
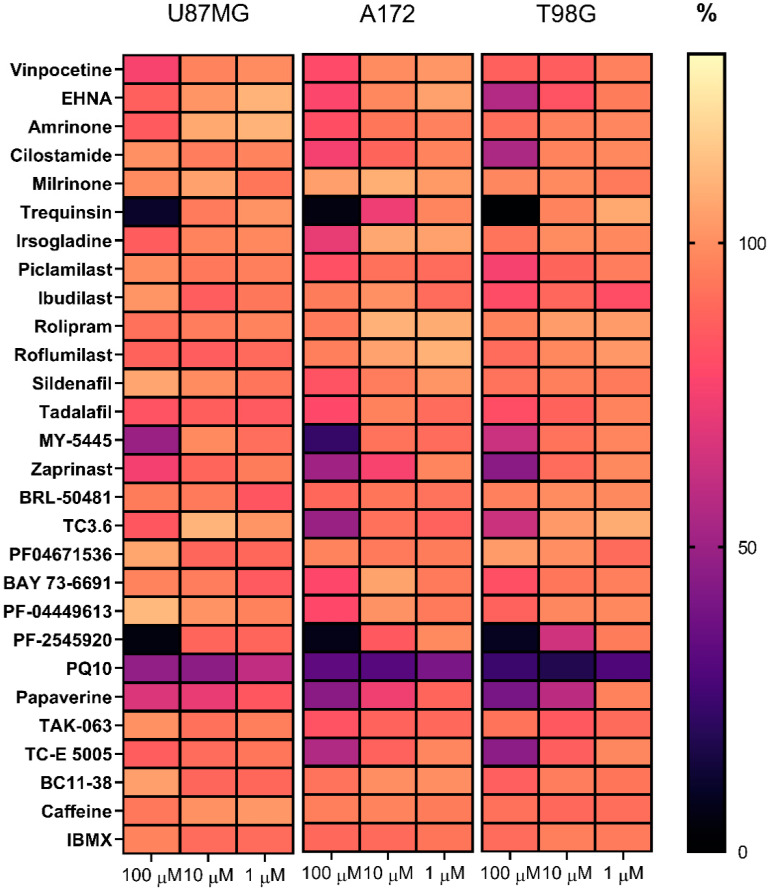
Antiproliferative activity of PDE inhibitors in glioblastoma cells. The heatmap illustrates the percentage of viable U87MG, A172 and T98G cells determined in the CCK-8 assay following treatment with PDE inhibitors at the indicated concentrations for 72 h (*n* = 3–12). Detailed quantitative data are presented in Supplementary Table S2.

To identify whichPDE inhibitors were able to attenuate the proliferation of U87MG, A172 and T98G glioblastoma cells, we tested 28 compounds inhibiting all the major PDE classes, using the CCK-8 viability assay (Figure 1). The most potent inhibitors of glioblastoma cell viability were the PDE10A inhibitors PF-2545920, PQ-10, papaverine and TC-E 5005, which decreased the percentage of viable cells by 55–95% in at least one of the three cell lines (Figure 1; Supplementary Table S2). Among the inhibitors of PDEs 1–4, only trequinsin had a pronounced antiproliferative effect, suppressing the number of viable cells in all three glioblastoma lines by 89–98.5%. Some inhibitors of PDEs 5–7, such as MY-5445, zaprinast and TC3.6, moderately inhibited cell viability by 51–77%. Other drugs, including all the tested inhibitors of PDEs 8, 9 and 11, suppressed the viability of the U87MG, A172 and T98G glioblastoma cells by not more than 20% (Figure 1; Supplementary Table S2). We observed that the U87MG cells were not as sensitive to PDE inhibitors as the A172 and T98G cells.

### 2.2. Effects of PDE Inhibitors on cAMP and cGMP Levels in Glioblastoma Cell Lines

**Figure 2:**
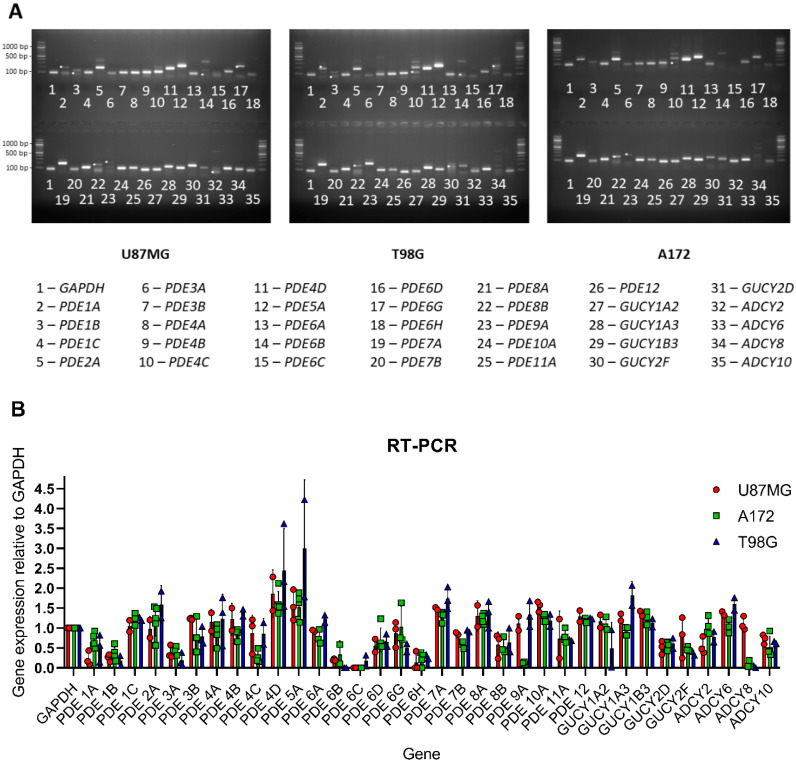
Relative expression levels of genes encoding PDEs, adenylyl cyclases and guanylyl cyclases in the U87MG, A172 and T98G glioblastoma cells. (**A**) Representative gels illustrating mRNA expression levels in each of the three glioblastoma lines tested. White asterisk (*) on the gel indicates the band of the correct size. (**B**) mRNA expression levels normalised by the *GAPDH* mRNA signal in glioblastoma cells grown to maximal confluence. Data are presented as the mean ± standard deviation of three independent experiments.

The relatively low antiproliferative potency of some PDE inhibitors was unlikely to be caused by the lack of expression of the corresponding PDE targets in glioblastoma cells, as our RT-PCR experiments revealed detectable transcription of the majority of PDEs in all three of the glioblastoma lines tested, except for some PDE6 isoforms (Figure 2). The relative mRNA expression levels of various PDEs were generally similar in all the lines, although the *PDE4C* and *PDE9A* mRNAs were appreciably expressed only in the U87MG and T98G cells, but not in the A172 cells. Likewise, the mRNA expression levels of enzymes generating cGMP and cAMP varied little in the three glioblastoma lines, although we noted that, unlike U87G cells, the A172 and T98G cells lacked the expression of the mRNA encoding adenylyl cyclase type 8.

**Figure 3:**
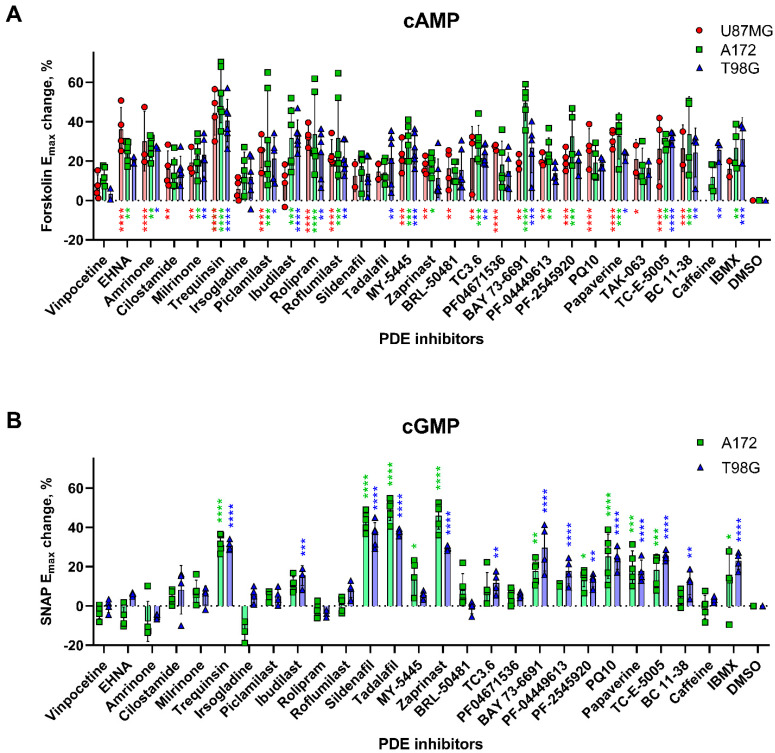
Effects of PDE inhibitors on cAMP and cGMP levels in glioblastoma cell lines. Incubation of glioblastoma cell lines with PDE inhibitors at a concentration of 100 μM altered cAMP (**A**) and cGMP (**B**) signals evoked, respectively, by 1 μM forskolin and 100 μM SNAP. Fractional changes are presented as the mean ± standard deviation of 4–6 independent experiments. The statistical significance of induced changes is indicated as follows: * *p* < 0.05; ** *p* < 0.01, *** *p* < 0.001, **** *p* < 0.0001.

The inhibition of cellular PDE activity should lead to an increase in the cyclic nucleotide concentration. We, therefore, determined the effects of PDE inhibitors on cAMP and cGMP levels in the glioblastoma cells (Figure 3). To increase the detectability of the signals associated with cyclic nucleotides, cAMP and cGMP levels in the cells were amplified, respectively, by a 30-min stimulation with 1 μM forskolin and a 10-min stimulation with 100 μM SNAP.

At a concentration of 100 μM, nearly all the tested PDE inhibitors, apart from vinpocetine, irsogladine and sildenafil, significantly increased cAMP levels in at least one of the three glioblastoma lines (Figure 3A). Fewer PDE inhibitors altered cGMP levels: only trequinsin, sildenafil, tadalafil and zaprinast increased cGMP levels by >25%, whereas lower, but still significant, increases in cGMP concentrations were caused by ibudilast, MY-5445, TC3.6, BAY 73-6691, PQ10, papaverine, PF-04449613, PF-2545920, TC-E 5005, BC 11-38 and IBMX in at least one of the two glioblastoma cell lines tested (Figure 3B).

Notably, although there were no significant correlations between the degree of elevation of cAMP and the extent of the antiproliferative effects of the inhibitors at a concentration of 100 μM, moderate negative correlations between the increase in cGMP level and cell survival were observed (Supplementary Figure S1). Generally, the drugs that strongly attenuated glioblastoma cell survival (Figure 1), such as trequinsin, MY-5445, PF-2545920 and PQ10, tended to significantly upregulate both cAMP and cGMP concentrations (Figure 3A,B). On the other hand, IBMX and ibudilast failed to cause appreciable antiproliferative effects, despite significantly enhancing both cGMP and cAMP levels (Figure 1 and Figure 3).

### 2.3. Effects of Combinations of PF-2545920 with Other PDE Inhibitors on the Survival of Glioblastoma Cells

**Figure 4:**
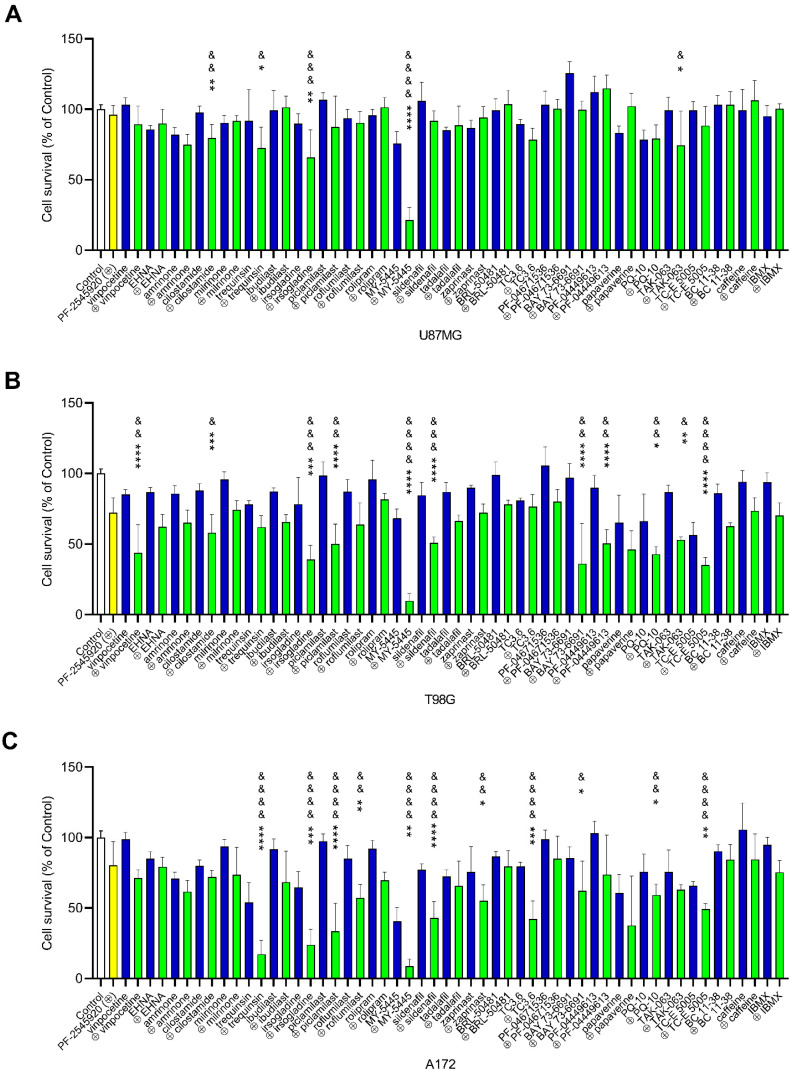
Effects of combinations of PF-2545920 with other PDE inhibitors on the survival of glioblastoma cells. CCK-8 proliferation assays were performed in the U87MG (**A**), T98G (**B**) and A172 (**C**) cell lines cultured with 10 μM PF-2545920 (yellow bar) with other PDE inhibitors used either at a concentration that inhibited cell viability by not more than 20% or at the default concentration of 100 μM (blue bars), or with combinations of these inhibitors at the same concentration with 10 μM PF-2545920 (green bars). Data are presented as the mean ± standard deviation. The statistical significance of differences in cell survival in the presence of drug combinations is indicated as follows: * *p* < 0.05; ** *p* < 0.01; *** *p* < 0.001; **** *p* < 0.0001 (from survival under PDE inhibitor alone); ^&^ *p* < 0.05; ^&&^ *p* < 0.01; ^&&&^ *p* < 0.001; ^&&&&^ *p* < 0.0001 (from survival under 10 μM PF-2545920).

Next, we examined whether combinations of PDE inhibitors would exert a synergistic suppressive effect on glioblastoma cell viability. We focused only on the combinations involving the PDE10A inhibitor PF-2545920 [21] because there is evidence of the high accumulation of this drug in the brain [22], and it was shown to be effective in colorectal cancer [23,24]. In an initial set of experiments, we performed the CCK-8 assay with glioblastoma cells treated with 10 μM PF-2545920, to which one other PDE inhibitor was added at a concentration, which, on its own, suppressed cell viability by no more than 20% (Figure 4). For weak inhibitors, the default concentration was 100 μM. We observed that a combination of 10 μM PF-2545920 and 50 μM MY-5445 had a particularly strong antiproliferative effect in all three lines tested (Figure 4A–C).

The additional combinations that led to at least a two-fold decrease in viability in at least one cell line included PF-2545920 and the PDE inhibitors irsogladine, piclamilast, trequinsin and sildenafil for A172 and T98G cells; vinpocetine, cilostimide, BAY-73-6691, PF04449613 and TAK-063 for A172 cells; and TC3.6 and papaverine for T98G cells (Figure 4A–C).

**Figure 5:**
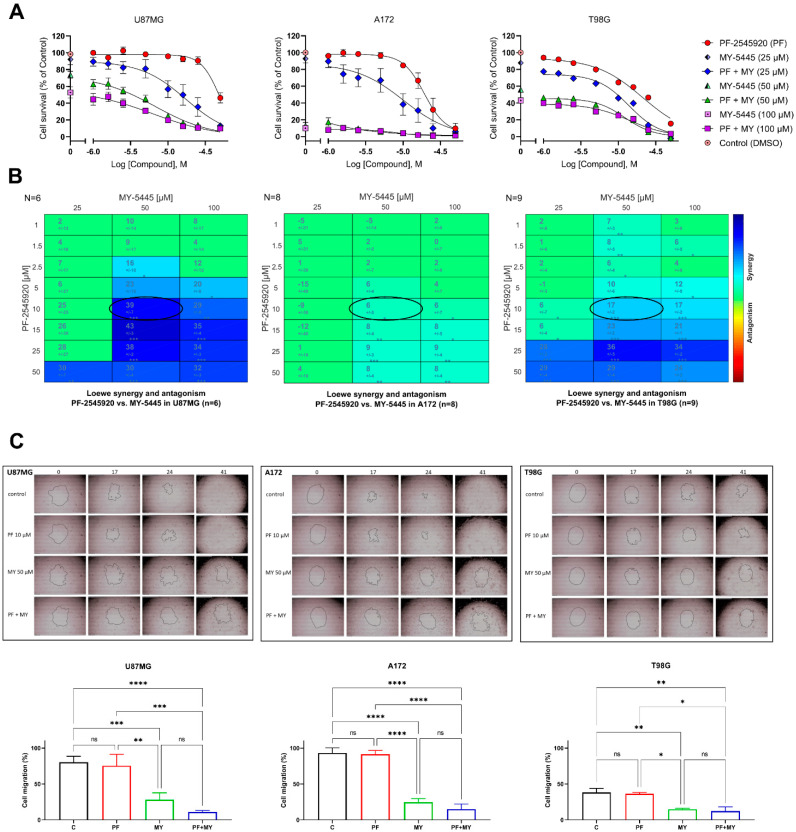
Synergistic suppressive action of PF-2545920 and MY-5445 on the viability and migration of glioblastoma cells. (**A**) Concentration–response relationships for the inhibitory action of PF-2545920 on the viability of U87MG, A172 and T98G cells were obtained using the CCK-8 assay in the absence or presence of MY-5445 at 25–100 μM. (**B**) In the synergy plots, the colour indicates the degree of synergism, and the values indicate synergy scores using the Loewe model as previously described [25]. The stronger the synergy is, the darker blue is the background, and the higher is the synergy score. Asterisks indicate the significance of synergy scores obtained following a one sample *t*-test (* *p* < 0.05; ** *p* <0.001, *** *p* < 0.0001; the number of replicates (N) is shown in the left top corner of the matrix display). (**C**) Migration capability of U87MG, A172 and T98G cells was assessed from the degree of closure of the initial cell-free gap area (outlined by a contour line in the centre of each image) after 24 h in culture using the Radius™ cell migration assay. C: control (vehicle); MY: 50 μM MY-5445; PF: 10 μM PF-2545920: PF+MY: combination treatment. Concentrations for the migration experiment were chosen from the synergy points designated by ovals in (**B**). Data are presented as the mean ± standard deviation (*n* = 3). The statistical significance of differences in cell migration is indicated as follows: * *p* < 0.05; ** *p* < 0.01; *** *p* < 0.001; **** *p* < 0.0001 (post hoc Tukey’s test).

Next, we determined the concentration–response relationships for the inhibitory effect of PF-2545920 on the survival of glioblastoma cells alone or when co-applied with MY-5445 at concentrations of 25, 50 and 100 μM (Figure 5A). We found that PF-2545920 and MY-5445 exerted a clear synergistic effect in the U87MG and T98G cell lines, whereas in A172 cells, synergy was not prominent, likely due to the overwhelming antiproliferative effect of 100 μM MY-5445 (Figure 5B).

As cell migration plays a pivotal role in cancer progression, we also determined the effect of the combination of PF-2545920 and MY-5445 on the migratory capacity of glioblastoma cell lines using the Radius™ cell migration assay. The U87MG and A172 cells that were treated by vehicle or 10 μM PF-2545920 achieved nearly a complete gap closure to 75–93% of the initial gap area in 24 h (Figure 5C). In contrast, the gap closure was significantly lower in the U87MG and A172 cells treated with 50 μM MY-5445 (27.98 and 24.56%, respectively) or with a combination of PF-2545920 and MY-5445 (10.90 and 14.86%, respectively). Qualitatively similar effects of these treatments were also observed in T98G cells, although in this case, the extent of the closure was lower for all the treatments, as even the vehicle-treated cells only covered 38.22% of the initial gap area after 24 h. In all three cell lines, the suppressive effect of the combination treatment on the degree of migration was significantly stronger than the effect of treatment with PF-2545920 alone (*p* < 0.05, post hoc Tukey test). The degrees of migration of cells treated with MY-5445 alone and with the combination of MY-5445 and PF-2545920 were not statistically different. However, in all three cell lines tested, maximal suppression was always achieved after the combination treatment.

### 2.4. Effects of Combinations of PF-2545920 and MY-5445 with MRP1 Inhibitors on the Survival of Glioblastoma Cells

**Figure 6:**
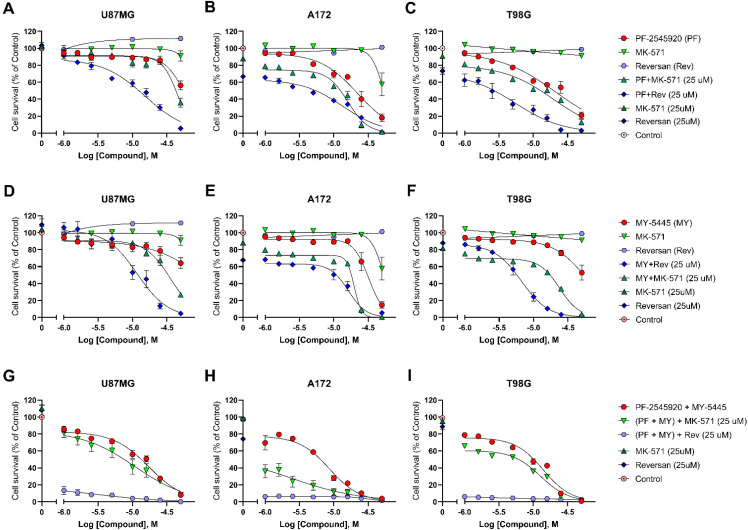
Potentiation of the antiproliferative action of PF-2545920 and MY-5445 in glioblastoma cells by the co-treatment with MRP1 inhibitors. Concentration–response relationships for the inhibitory action of PDE inhibitors PF-2545920 (**A**–**C**) and MY-5445 (**D**–**F**) on glioblastoma cell survival were obtained using the CCK-8 assay in the absence or presence of the MRP1 inhibitors MK-571 and reversan. Maximal antiproliferation effects were observed when either MK-571 or reversan was co-applied with both PDE inhibitors (**G**–**I**). Data are presented as the mean ± standard error of the mean (*n* = 6–12).

Since, on the one hand, MRPs affect the intracellular concentrations of cAMP and cGMP [16] and, on the other hand, MRP inhibitors were shown to sensitise glioblastoma cells to the cytotoxic action of other drugs [19,20], we investigated the effect of treatments with PF-2545920 and/or MY-5445 in combination with MRP1 inhibitors MK-571 and reversan on glioblastoma cells (Figure 6).

Incubation with either MK-571 or reversan alone at a concentration range 1–50 μM did not appreciably affect the survival of the U87MG and T98G cells, although in the A172 cells, treatment with MK-571 at a high concentration of 50 μM decreased the number of viable cells to 58% of the control (Figure 6B). Co-treatment with 25 μM reversan significantly potentiated the antiproliferative effects of both PF-2545920 (Figure 6A–C) and MY-5445 (Figure 6D–F). Furthermore, the incubation of all three glioblastoma lines with PF-2545920, MY-5445 and 25 μM reversan simultaneously caused a particularly strong suppression of glioblastoma cell growth; therefore, the number of viable cells was decreased by >80%, even at low concentrations of PF-2545920 and MY-5445 (Figure 6G–I). MK-571 also potentiated the antiproliferative action of the PDE inhibitors upon co-treatment, although to a lower degree in the A172 (Figure 6B,E,H) and T98G cells (Figure 6C,F,I). In the U87MG cells, a significant potentiating effect of 25 μM MK-571 was observed only during co-treatment with the highest tested concentration of MY-5445 (50 μM, Figure 6D).

## 3. Discussion

It has been known since the 1970s that brain tumours, and glioblastomas in particular, have lower concentrations of cAMP and cGMP than normal brain tissue [26,27,28,29]. PDEs catalyse the hydrolysis of cyclic nucleotides and are, therefore, important physiological regulators of cAMP and cGMP levels [30]. The inhibition of PDEs has been proposed as a viable therapeutic strategy in many cancers [7,31]. Given that multiple PDE isoforms are expressed in the brain [32,33], we screened 28 compounds that inhibit all the major PDEs and identified several inhibitors with promising antiproliferative activity against the U87MG, A172 and T98G glioblastoma cell lines. Further experiments using the PDE10A inhibitor PF-2545920 and the PDE5 inhibitor MY-5445 revealed the synergistic suppression of proliferation in two of the three glioblastoma cell lines tested. Finally, we demonstrated that treatment with PF-2545920 and MY-5445 at low micromolar concentrations in combination with the MRP1 inhibitor reversan afforded nearly complete inhibition of glioblastoma cell growth.

Our present results broaden the spectrum of PDE inhibitors that might be used to attenuate the proliferation and survival of glioblastoma cells. Several previous studies have used pharmacological inhibition of PDE4 by rolipram to increase cAMP concentrations and thereby decrease the viability of glioblastoma cells [9,34]. In our experiments, 100 μM rolipram did not have a pronounced inhibitory effect on the survival of glioblastoma cells, but this result is not necessarily in disagreement with previous reports, as potent suppressive activity against U87MG and A172 cells has only been reported at several-fold higher concentrations of the drug, up to 1 mM [34], or when rolipram was combined with forskolin [9]. However, we did observe a modest antiproliferative effect of the PDE4 inhibitors irsogladine and piclamilast, particularly when they were combined with the PDE10A inhibitor PF-2545920. In addition, we observed a moderate effect of the PDE1 inhibitor vinpocetine at a concentration of 100 μM, which was broadly in agreement with the published data on its antiproliferative action in both the U251MG cells and several primary glioblastoma tumour cell lines (IC_50_ = 34–273 μM) [13]. Further, our data regarding the effect of the PDE10 inhibitor papaverine are in line with those of Inada et al., who showed the antiproliferative action of papaverine against U87MG and T98G cells in the WST-8 assay with IC_50_ values of 29 and 40 μM, respectively [35].

The inhibition of glioblastoma cell proliferation has also been achieved by treatment with relatively non-selective PDE inhibitors, such as the methylxanthines theophylline, theobromine, caffeine and IBMX [9,36,37,38,39,40]. In general, these compounds decrease glioblastoma cell survival at concentrations above 100 μM, which is similar to the low potency of caffeine and IBMX observed in our experiments (<10% suppression of proliferation at 100 μM). Another non-selective PDE inhibitor, zaprinast, was reported to block the proliferation of U87 cells by ~20% at a concentration of 100 μM [37]. This was also seen in our experiments, where zaprinast had a comparable effect on U87MG cells, while its antiproliferative action against A172 and T98G cells was even stronger. Thus, our screening of several previously tested PDE inhibitors was largely consistent with the results of published reports. However, to the best of our knowledge, this is the first report of potent suppression of human glioblastoma cell viability by the compounds trequinsin, MY-5445, PF-2545920, PQ10 and TC-E-5005.

Notably, although all the PDE inhibitors with high antiproliferative potency in our experiments caused the elevation of both the cAMP and cGMP concentrations, a general increase in the cyclic nucleotide levels on their own was likely a necessary but not sufficient condition for suppressing glioblastoma cell proliferation, because several PDE inhibitors failed to affect cell survival despite they increased cAMP and cGMP levels. In our recent study in rat glioma cells, a significant correlation was found between the ability of PDE inhibitors to suppress cell growth and elevate cAMP (but not cGMP) levels [41]. However, we note that the effects of the drugs on cell survival were quite different in rat glioma cells compared to those in the human glioblastoma cells in the present study.

The pronounced antiproliferative action of some of the PDE inhibitors on glioblastoma cells may be partially explained by their additional effect on non-PDE targets. For example, zaprinast is a strong glutaminase inhibitor that may decrease the abnormally high levels of D-2-hydroxyglutarate in glioblastoma cells [42]. Papaverine is a mitochondrial C1 inhibitor, which may account for its radiosensitising action in solid tumours [43], while MY-5445 has been reported to antagonise the ABCG2 transporter, the upregulation of which is implicated in cancer multidrug resistance [44].

Drug combinations comprising PDE inhibitors have been used to treat several cancers, including glioblastoma. In particular, rolipram, ibudilast and papaverine have been shown to augment the effects of temozolomide, the current standard of care in glioblastoma treatment [45,46,47]. Sildenafil is currently in clinical trials for the treatment of recurrent high-grade glioma in combination with sorafenib and valproic acid [48]. Interestingly, the inclusion of ibudilast, papaverine and sildenafil in such combinations is not based mainly on their inhibitory efficacy against respective PDEs. Papaverine potently disrupted the interaction between high mobility group box 1 (HMGB1) and the receptor for advanced glycation end-products (RAGE) [49], which was thought to be the essential mechanism of its anti-proliferative action against glioblastoma cells [35,46]. Ibudilast sensitised glioblastoma cells to temozolomide by inhibiting the macrophage migration inhibitory factor [47], while sildenafil is included on the basis of its ability to inhibit drug transporters ABCB1 and ABCG2 [50]. Our experiments showed the potent antiproliferative actions of several previously untested combinations of PDE inhibitors, including the synergism of the effects of PF-2545920 and MY-5445. It is possible that the particular efficacy of this combination could, again, be partly explained by the inhibitory action of MY-5445 on the ABC family drug transporters [44].

Migration and invasion are crucial cellular mechanisms in the spread of glioblastoma within the brain. Several PDE inhibitors have been tested for their action on the migratory and invasive properties of cancer cells. IBMX (500 µM), dipyridamole (100 µM), milrinone (10 µM), piclamilast (1 µM), rolipram (10 µM), the PDE7 inhibitor spiroquinazolinone (1 µM) and the PDE8 inhibitor PF-04957325 (1 µM) significantly attenuated the migration of MDA-MB-231 breast cancer cells in transwell and/or wound healing assays [51]. The PDE5 inhibitors vardenafil and sildenafil, at a concentration of 100 µM, also significantly suppressed the migration of prostate cancer cell lines in the wound healing assay [52]. At the same concentration, IBMX and cilostamide inhibited the migration of Panc1 and MiaPaCa2 pancreatic cancer cells [53]. Caffeine at concentrations of 100 and 500 µM inhibited the migration of U87MG cells [38], whereas vinpocetine (30–300 µM) failed to significantly affect the cell migration of primary glioblastoma cells [13]. Interestingly, at a relatively low concentration (1 µM), sildenafil *increased* the wound-healing ability of T98G cells, a potentiating effect that was not seen in cells after PDE5 knockdown [14]. In our experiments, the PDE5 inhibitor MY-5445 at a concentration of 50 μM had a clear inhibitory effect on the migration of all three glioblastoma lines tested, including T98G cells, which was further potentiated by the co-application of 10 μM PF-02545920. The qualitatively opposite actions of the PDE5 inhibitors sildenafil [14] and MY-5445 (this study) on cell migration could be explained by the different potencies of the drugs, distinct effects on other targets [44], or differences in the assays used. Further experiments using the invasion assay will be required to confirm the ability of PDE inhibitors to prevent the spread of glioblastoma cells.

The maximal suppression of glioblastoma cell viability in our experiments was achieved by treatment with drug combinations that included the MRP1 inhibitor reversan. Co-treatment with another MRP1 inhibitor, MK-571, led to qualitatively similar but less dramatic effects. Although neither MRP1 inhibitor alone significantly affected the proliferation of glioblastoma cells at 25 µM, they significantly enhanced the antiproliferative actions of PF-2545920 and MY-5445, particularly in ternary combinations. Although MRP proteins may take part in the regulation of cAMP and cGMP levels [16], it is more likely that the additive negative effect of MRP1 inhibitors on glioblastoma cell proliferation in our experiments was linked to the inhibition of PDE inhibitor efflux. The up-regulation of MRP1 expression underlies the multidrug resistance mechanisms in various tumours, including high-grade gliomas, as well as glioblastoma cell lines, such as T98G [18,54,55,56]. Various MRP1 inhibitors have been shown to increase the efficacy of conventional chemotherapies in glioblastoma cells [17,18,19,20].

Our present results allow a cautious conclusion that the potent inhibition of glioblastoma cell viability may be achieved by combining MRP1 inhibitors with PDE inhibitors, especially the PDE10A inhibitor PF-2545920. The next logical steps in the translational preclinical evaluation of PDE inhibitors and their combinations may involve in vivo experiments in PDX mice and in vitro tests in organoids from patient-derived glioblastoma cells. Such studies, as well as a further elaboration of the molecular mechanisms and signalling pathways mediating the antiproliferative effect of the tested combinations of PDE and MRP1 inhibitors, will be necessary before assessing the potential efficacy of these treatments in the clinical setting.

## 4. Materials and Methods

### 4.1. Cell Culture and Reagents

The human glioblastoma astrocytoma cell lines U87MG (ECACC 89081402), derived from a female patient with grade IV glioma [57], and A172 (ECACC 88062428), derived from a 53-year-old male with glioblastoma [58], were obtained from the European Collection of Authenticated Cell Cultures. The T98G cell line, derived from a glioblastoma removed from a 61-year-old male [59], was obtained from the American Type Culture Collection (ATCC^®^ CRL1690™). Mutations in multiple genes, including *TP53* (for T98G), *PTEN* (for T98G), *RB1* (for A172), *NF1* (for U87MG, T98G) and others, have been reported previously in these cell lines [60]. All cell lines were maintained in Dulbecco’s Modified Eagle Medium/Nutrient Mixture F-12 (Gibco, Thermo Fisher Scientific, Paisley, UK) supplemented with 10% foetal bovine serum (Merck Life Science, Gillingham, UK) and 5% antibiotic antimycotic solution (10,000 units penicillin, 10 mg streptomycin and 25 μg/mL amphotericin B; Merck Life Science, Gillingham, UK) at 37 °C in the humidified atmosphere of 95% air and 5% CO_2_. 

The following twenty-two previously reported PDE inhibitors were purchased from Sigma (Gillingham, UK): vinpocetine (PDE1), EHNA (PDE2), amrinone, cilostamide, milrinone (all PDE3), trequinsin (PDE3/4), ibudilast, piclamilast, roflumilast, rolipram (all PDE4), sildenafil, tadalafil (all PDE5), zaprinast (PDE5/6), BRL-50481, TC3.6 (all PDE7), BAY 73-6691, PF-04449613 (all PDE9), PF-2545920, PQ-10 (all PDE10A), BC 11-38 (PDE11), caffeine and IBMX (non-selective cAMP PDE inhibitors). Four PDE inhibitors, irsogladine (PDE4), MY-5445 (PDE5), PF-04671536 (PDE8) and TC-E 5005 (PDE10A), were purchased from Tocris Bioscience (Bristol, UK). Two PDE10A inhibitors, papaverine and TAK-063, were from Acros Organics (Geel, Belgium) and Selleckchem (München, Germany), respectively. The MRP1 inhibitors reversan and MK-571 were purchased from Sigma. All compounds were dissolved in dimethyl sulfoxide (DMSO) to obtain 10–100 mM stock solutions, except papaverine and MK-571, which were dissolved in sterile water.

### 4.2. Cell Viability Assay

Cell viability was measured using the Cell Counting Kit-8 (CCK-8) assay (Sigma, Gillingham, UK). U87MG, A172 and T98G cells were seeded at a density of 8,000 cells/well in 96-well plates and allowed to adhere overnight at 37 °C in a humidified atmosphere of 95% air and 5% CO_2_. No cells were seeded in the perimeter wells to ensure measurement accuracy and those were filled with 100 μL of sterile water. Initially, all compounds were tested at the following three concentrations: 100, 10 and 1 μM. All initial dilutions were made in DMSO (except for papaverine, which was diluted in sterile water) and then diluted in the medium at 1:100 ratio. Compounds that showed antiproliferative activity were tested at additional concentrations to generate dose–response curves. Then, culture medium was removed from the plates, and fresh medium containing tested compounds at different dilutions was added. Control cells were treated with vehicle solution containing 1% DMSO or 1% sterile water. Blank controls without cells were also prepared. At 72 h after treatment, 5 μL of the CCK-8 solution was added to every well containing 100 μL of tested compounds, controls or blank. After 3 h of incubation at 37 °C in the dark, the plates were read using a Mithras LB940 multimode microplate reader (Berthold Technologies, Harpenden, UK), and the absorbance values were determined at 490 nm. The percentage of viable cells was calculated for each well as follows: % cell survival = {(A_t_ − A_b_)/(A_c_ − A_b_)} × 100, where A_t_ is absorbance of the medium with tested compound, A_c_ is absorbance of control medium and A_b_ is absorbance of blank medium.

### 4.3. cAMP Accumulation Assay

Cells were trypsinised, washed in phosphate-buffered saline (PBS) and resuspended in the stimulation buffer (PBS containing 0.1% bovine serum albumin). The number of cells per well was optimised to obtain the best response for each cell line and comprised 7000, 1000 and 800 for the A172, U87MG and T98G cell lines, respectively. Cells were added to 384-well white optiplates (PerkinElmer Life & Analytical Sciences, Seer Green, UK). The compounds were diluted in a 96-well plate in the stimulation buffer containing 1 μM forskolin and added at concentrations ranging between 100 nM and 100 μM. The cAMP accumulation was measured after 30-min stimulation using a LANCE cAMP detection kit (PerkinElmer Life & Analytical Sciences, Seer Green, UK). Plates were read using a Mithras LB940 multimode microplate reader (Berthold Technologies, Harpenden, UK). Percentages of the maximum response (E_max_) change in cells after forskolin stimulation were calculated for each PDE inhibitor using the following formula: %E_max_ change = 100 − (A_t_/A_c_ × 100), where E_max_ is efficacy or the maximum response, A_t_ is absorbance of the stimulation buffer with tested compound and A_c_ is absorbance of stimulation buffer with DMSO.

### 4.4. cGMP Accumulation Assay

Cells were trypsinised and resuspended in the stimulation buffer (PBS containing 0.1% bovine serum albumin). The optimal numbers of cells per well, which generated a signal within the linear range of the standard curve, were 20,000 for A172 cells and 40,000 for T98G. We found it problematic to optimise the cGMP assay for the U87MG cell line, because over 80,000 cells per well were required; therefore, this line was not included in this experiment. The cells were pre-treated with compounds at a concentration of 100 μM and added to 384-well white optiplates (PerkinElmer Life & Analytical Sciences, Seer Green, UK). cGMP was detected after a 10-min stimulation using the cGMP assay based on the HTRF^®^ technology (Cisbio Bioassays, Codolet, France). The NO donor (S)-nitroso-N-acetylpenicillamine (SNAP; Tocris, Bristol, UK), an activator of soluble guanylate cyclase, was dissolved in DMSO and diluted in the stimulation buffer at 0.1–100 µM in 96-well plates, which were read using a Mithras LB940 multimode microplate reader (Berthold Technologies, Harpenden, UK). The following ratio of the acceptor and donor emission signals was calculated for each well: Ratio = Signal_665nm_/Signal_620nm_ × 10,000.

The percentage of the E_max_ change in cells after SNAP stimulation was calculated for each PDE inhibitor and analysed in the same way as in the cAMP accumulation assay.

### 4.5. Gene Expression

U87MG, T98G and A172 cells were seeded in T75 flasks and allowed to grow to maximal confluence. The cells were trypsinised, and total RNA was extracted using an RNeasy Mini kit (Qiagen, Manchester, UK) according to the manufacturer’s instructions. A NanoDrop Lite spectrophotometer (Thermo Fisher Scientific, Paisley, UK) was used for measurements of RNA quantity and purity. Aliquots of RNA were frozen at −20 °C for the subsequent study of the expression levels of genes encoding PDEs, guanylyl cyclases and adenylyl cyclases by using RT-PCR with a QuantiTect reverse transcription kit (Qiagen, Manchester, UK). The primers used for PCR amplification are indicated in Supplementary Table S1. All PCR products were resolved on a 2% agarose gel with ethidium bromide and imaged using a G:Box iChemi gel documentation system (Syngene, Cambridge, UK). The density of each band was analysed with GeneTools software (Syngene, Cambridge, UK). Densitometry measurements of each gene were normalised using the *GAPDH* mRNA signal.

### 4.6. Drug Combination Assays

To study the effects of combined treatments of the PDE10A inhibitor PF-2545920 with other PDE inhibitors, the CCK-8 viability assay was performed as described above. Initially, a combination of 10 μM PF-2545920 and another PDE inhibitor at a concentration at which the inhibition of glioblastoma cell viability was no more than 20% or, if virtually no inhibition was seen, at 100 μM. Thus, 10 μM PF-2545920 was co-applied with PQ-10 at 0.25 μM; with trequinsin at 25 μM; with MY-5445, zaprinast, TC-E 5005, papaverine and TC3.6 at 50 μM; and with the remaining PDE inhibitors at 100 μM. Then, concentration–response relationships for PF-2545920 were determined in the presence of MY-5445, co-treatment with which, in the previous experiment, suppressed viability by >50% in all three glioblastoma cell lines.

### 4.7. Cell Migration Assay

To study their migratory properties, glioblastoma cells were plated at a density of 8000 cells/well into a Radius^TM^ 96-well cell migration assay plate (Cell Biolabs, Inc., San Diego, CA, USA) and allowed to adhere overnight at 37 °C in a humidified atmosphere of 95% air and 5% CO_2_. Each plate well contained a 0.68-millimeter non-toxic, biocompatible hydrogel spot, where the cells cannot attach. At the start of the experiment, the gel spot was removed according to the manufacturer’s instructions, and fresh medium containing tested compounds at different concentrations added. Control cells were treated with the medium containing 1.1% DMSO. Digital images of the gap closure were taken with a DinoEye Edge 5MP eyepiece digital camera (Lambda Photometrics Ltd., Harpenden, UK) and analysed with ImageJ software.

### 4.8. Statistical Analysis

Cell survival data were normalised to the average signal from control wells (100%) and the statistical significance of differences from control values was assessed using the one sample *t*-test (in comparison to the hypothetical mean of 100%). Concentration–effect relationships for drug inhibitor assays were analysed by using Prism 8 (GraphPad, Inc., San Diego, CA, USA). Data were fitted by the four-parameter logistic equation to obtain pIC_50_ values. The analysis of combination effects was performed using Combenefit software (version 2.021) [25] with the additive Loewe synergy effect as a baseline model [61,62]. Cell migration was calculated as the difference between the initial area of the hydrogel spot in the beginning of the experiment and the area that remained free of cells in 24 h. Data are presented as the mean ± standard deviation. Statistical significance of the drug effects was analysed using the Student’s *t*-test for pairwise comparisons or by one-way analysis of variance followed by the Dunnett’s or Tukey’s post hoc tests for comparisons involving more than two groups. Correlation between the extent of the antiproliferative effect of PDE inhibitors and the degree of elevation of cAMP or cGMP levels was assessed by calculating Pearson coefficient *r*.

## Data Availability

All data generated during this study are included in this published article and Supplementary Material.

## Supplementary Materials

The following are available online at https://www.mdpi.com/article/10.3390/ijms22189665/s1, Figure S1: Analysis of the relationships between the degree of elevation of cAMP or cGMP levels and the extent of the antiproliferative effect of PDE inhibitors at a concentration of 100 μM in glioblastoma cell lines U87MG, A172 and T98G, Table S1: Primers used for PCR, Table S2: Details of statistical analysis of the experiments illustrated in Figure 1.
