# Supplementary material for: Suppression of Proliferation of Human Glioblastoma Cells by Combined Phosphodiesterase and Multidrug Resistance-Associated Protein 1 Inhibition
Source: Int J Mol Sci. 2021 Sep 7;22(18):9665. doi: 10.3390/ijms22189665 (PMC8471536; doi:10.3390/ijms22189665)
Supplement: Supplementary file 1 [file ijms-22-09665-s001.zip › Supplementary Figure S1.pdf]

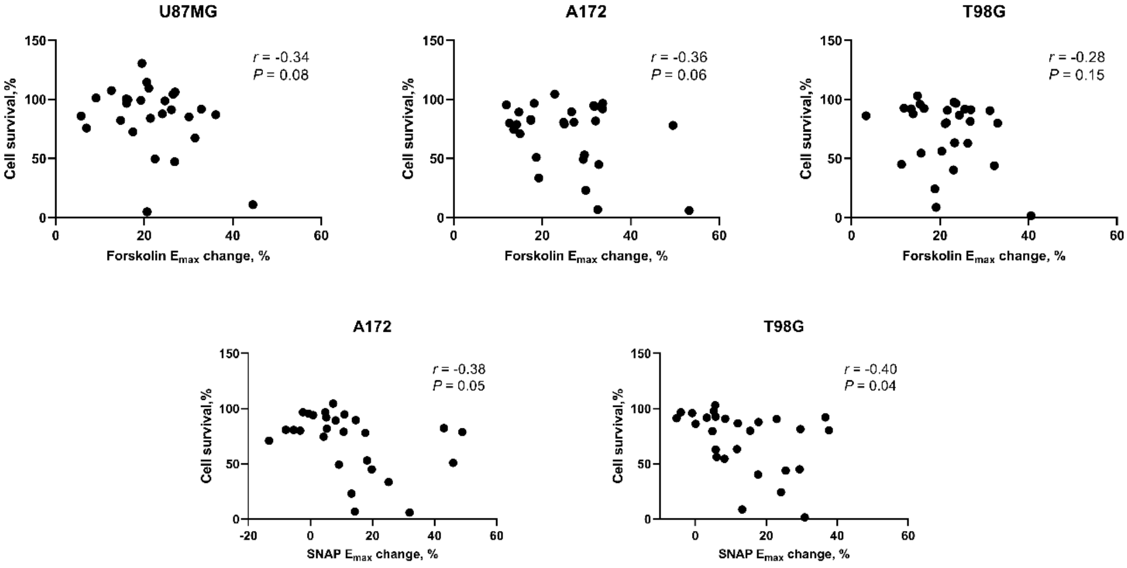

Supplementary Figure S1. Analysis of the relationships between the degree of elevation of cAMP or cGMP levels and the extent of the antiproliferative effect of PDE inhibitors at a concentration of 100 µM in glioblastoma cell lines U87MG, A172 and T98G. Changes in the level of cAMP and cGMP were inferred from the percentages of the maximum response (E_{max}) change in cells after stimulation with forskolin (upper row) and SNAP (lower row), respectively. Each point represents an individual PDE inhibitor tested. Pearson correlation coefficient *r* and two-tailed *p*-value are indicated.
