# Supplementary material for: Suppression of Proliferation of Human Glioblastoma Cells by Combined Phosphodiesterase and Multidrug Resistance-Associated Protein 1 Inhibition
Source: Int J Mol Sci. 2021 Sep 7;22(18):9665. doi: 10.3390/ijms22189665 (PMC8471536; doi:10.3390/ijms22189665)
Supplement: Supplementary file 1 [file ijms-22-09665-s001.zip › Supplementary Material.pdf]

Supplementary Material Description

Kopanitsa et al. "*Suppression of proliferation of human glioblastoma cells by combined phosphodiesterase and multidrug resistance-associated protein 1 inhibition*"

Supplementary Table S1. Primers used for PCR.

Supplementary Table S2. Details of statistical analysis of the experiments illustrated in Figure 1.

Supplementary Figure S1. Analysis of the relationships between the degree of elevation of cAMP or cGMP levels and the extent of the antiproliferative effect of PDE inhibitors at a concentration of 100 μM in glioblastoma cell lines U87MG, A172 and T98G. Changes in the level of cAMP and cGMP were inferred from the percentages of the maximum response (E_{max}) change in cells after stimulation with forskolin (upper row) and SNAP (lower row), respectively. Each point represents an individual PDE inhibitor tested. Pearson correlation coefficient r and two-tailed P-value are indicated.
