# Supplementary material for: Suppression of Proliferation of Human Glioblastoma Cells by Combined Phosphodiesterase and Multidrug Resistance-Associated Protein 1 Inhibition
Source: Int J Mol Sci. 2021 Sep 7;22(18):9665. doi: 10.3390/ijms22189665 (PMC8471536; doi:10.3390/ijms22189665)
Supplement: Supplementary file 1 [file ijms-22-09665-s001.zip › Supplementary Table S1.pdf]

Supplementary Table S1. Primers used for PCR.

N	Gene	Sequence (5′ → 3′)	Amplicon Size (bp)	PrimerBank or PubMed ID	N	Gene	Sequence (5′ → 3′)	Amplicon Size (bp)	PrimerBank or PubMed ID
**1**	*GAPDH*	F: TGCACCACCAACTGCTTAGC<br>R: GGCATGGACTGTGGTCATGAG	87	PMID: 12184808	**19**	*PDE7A*	F: CGTATGCTAGGAGATGTACGTGT<br>R: TGAAACCGCAGTACCACGAAA	201	341823662c1
**2**	*PDE1A*	F: ATGGGGTCTAGTGCCACAGAG<br>R: GCACAGATGCCGCATATTCAAT	187	51102298c1	**20**	*PDE7B*	F: GGTTGAGAGGTGTGGCGAAAT<br>R: CCTTAGTCGTATATCTCCCAGCA	85	57242789c1
**3**	*PDE1B*	F: CTGCGCTACATGGTGAAGCA<br>R: CAAGATTTGCCGTGTCTCATCTA	123	260436978c1	**21**	*PDE8A*	F: TCCAGCCAGAGACGACACT<br>R: ACAGGCATGGGACTACTTTCC	116	341823697c2
**4**	*PDE1C*	F: ATAAGACAGGAGTGGCGAACT<br>R: GTATGCTCGTAGTCATGGATGG	85	300796870c3	**22**	*PDE8B*	F: ACGCAGGCTTCAACAGGAG<br>R: CGTGGTCATCGCTTGTTATTTCT	177	300244576c3
**5**	*PDE2A*	F: GACCGCAAGATCCTCCAACTG<br>R: CCGAGCACTTTGTCTCCGA	176	344925850c2	**23**	*PDE9A*	F: GACTCCTCGACGCGATGTTC<br>R: TTTCTGTAGTTGTCGTGGACG	222	48762733c1
**6**	*PDE3A*	F: CCACGGCCTCATTACCGAC<br>R: TTGCTCACGGCTCTCAAGG	78	347658968c1	**24**	*PDE10A*	F: GAGACAACCAGCTACTCCTCT<br>R: ACAGGCTATTATTGCACTCTCCA	105	359465520c2
**7**	*PDE3B*	F: GTGCCGCCGAAGAAAAAGTG<br>R: CAACTGCCATAGTAACTGGCTG	94	219879808c1	**25**	*PDE11A*	F: TGATGACTTTTCTCTCGACGTTG<br>R: AAGCCACCTACACAGTGTCTC	115	116536086c1
**8**	*PDE4A*	F: ACACACCTGTCAGAAATGAGC<br>R: GGGTGATGGGATCTCCACTTC	99	341572546c1	**26**	*PDE12*	F: GTAGTGCGCTGCGTACCTT<br>R: GCGCTGCATGTTCTTGTGG	81	189027128c1
**9**	*PDE4B*	F: AACGCTGGAGGAATTAGACTGG<br>R: GCTCCCGGTTCAGCATTCT	110	82799485c1	**27**	*GUCY1A2*	F: GAGGACTGCTTCGAGATTGTATC<br>R: TGGTTCTAATCACAAACGGGGTA	91	374093197c3
**10**	*PDE4C*	F: GAGACGCTAGACGAGCTGGA<br>R: GTGGGTCAACTCCCGGTTC	120	341604763c2	**28**	*GUCY1A3*	F: AGTGAGGAGATGGGACAACTC<br>R: GAGCCCTCGTCCTGTAAAATC	148	374253805c3
**11**	*PDE4D*	F: GACCAATGTCTCAGATCAGTGG<br>R: GTCAAGGGCCGGTTACCAG	187	308387383c1	**29**	*GUCY1B3*	F: CTGCTTACGTCTCAAGGGTCA<br>R: ATGTCACTTAGATACAGCCCTCT	123	221139770c3
**12**	*PDE5A*	F: CGGCCTCTTAGACCCATTGTT<br>R: AGGGAATAGCGGTCAGCAGAT	239	61744429c2	**30**	*GUCY2F*	F: GAGGTTGCTGCGCGATTAG<br>R: CCAGGGTTGGTAGGTCCAATAA	173	134152693c2
**13**	*PDE6A*	F: CATCGAACGACAGTTCCACAA<br>R: GGTCATGTCTAAGAGACCCACAG	88	170650673c3	**31**	*GUCY2D*	F: TCGTGCTGGTTCGATCCAAAC<br>R: GTAGCCGGTGCCTCACATAAT	133	169791019c3
**14**	*PDE6B*	F: ACACCCTCAGCCGATCACT<br>R: ACATTTCGTCAGCGGAAGCAT	103	223718033c2	**32**	*ADCY2*	F: GGGACCAGGTATCGTTCTTCC<br>R: GTGAGGACGCTGGCAATGAT	94	115387101c2
**15**	*PDE6C*	F: ATGGACAAGCAAACTGGGTATG<br>R: TGGTGTGATGAAGCCTTAGGAT	187	57364938c2	**33**	*ADCY6*	F: CTCCTGGTCCCTAAAGTGGAT<br>R: GGAGGCAGCTCATATAGCGG	124	168480141c1
**16**	*PDE6D*	F: TTCAAAGGGCAATGCCTAGAAG<br>R: TTCCCAGTTAAGACGCTTGCT	134	56676309c3	**34**	*ADCY8*	F: CCTGCGGCACCAAAGTCTT<br>R: CGAGTTGCTAGGGGCACAG	110	168480145c1
**17**	*PDE6G*	F: AAGGCTAGAGATGACCTCGG<br>R: GGGGTCCCTAAGTGAAAAGC	246	PMID: 20655036	**35**	*ADCY10*	F: GTGACGATTGTGTTTGTGAACC<br>R: GATGTGCATATAGGCATCCTGG	90	268370068c3
**18**	*PDE6H*	F: GCAGACTCGCCAATTCAAGAG<br>R: TCTGTTCCTAGCCCCTCCATT	90	291045288c1					
